# Lipodystrophy diagnosis in people living with HIV/AIDS: prediction and validation of sex-specific anthropometric models

**DOI:** 10.1186/s12889-018-5707-z

**Published:** 2018-06-27

**Authors:** André P. dos Santos, Anderson M. Navarro, Andiara Schwingel, Thiago C. Alves, Pedro P. Abdalla, Ana Claudia R. Venturini, Rodrigo C. de Santana, Dalmo R. L. Machado

**Affiliations:** 1 Interunit Nursing Doctoral Program, College of Nursing of the University of Sao Paulo, Avenida dos Bandeirantes, Campus Universitario, 3900, Ribeirao Preto, SP 14040-902 Brazil; 2 Department of Clinical Medicine, Faculty of Medicine at the University of Sao Paulo, Ribeirao Preto, SP Brazil; 3 Kinesiology and Community Health, University of Illinois, Urbana-Champaign, IL USA; 4 School of Physical Education and Sport of Ribeirao Preto, University of Sao Paulo, Ribeirao Preto, SP Brazil

**Keywords:** Anthropometry, Body composition, Lipoatrophy, Lipohypertrophy, cART

## Abstract

**Background:**

Body composition alterations, or lipodystrophy, can lead to serious health problems in people living with HIV/AIDS (PLWHA). The objectives of this study are to predict and validate sex-specific anthropometric predictive models for the diagnosis of lipodystrophy in PLWHA.

**Methods:**

A cross-sectional design was employed to recruit 106 PLWHA (me*n* = 65 and women = 41) in Brazil during 2013–2014. They were evaluated using dual-energy X-ray absorptiometry, and 19 regions of body perimeters and 6 skinfold thicknesses were taken. Sex-specific predictive models for lipodystrophy diagnosis were developed through stepwise linear regression analysis. Cross-validations using predicted residual error sum of squares was performed to validate each predictive model.

**Results:**

Results support the use of anthropometry for the diagnosis of lipodystrophy in men and women living with HIV/AIDS. A high power of determination with a small degree of error was observed for lipodystrophy diagnosis for men in model six (r^2^ = 0.77, SEE = 0.14, r^2^_PRESS_ = 0.73, SEE _PRESS_ = 0.15), that included ratio of skinfold thickness of subscapular to medial calf, skinfold thickness of thigh, body circumference of waist, formal education _years_, time of diagnosis to HIV _months_, and type of combined antiretroviral therapy (cART) (with protease inhibitor “WI/PI = 1” or without protease inhibitor “WO/PI = 0”); and model five for women (r^2^ = 0.78, SEE = 0.11, r^2^_PRESS_ = 0.71, SEE _PRESS_ = 0.12), that included skinfold thickness of thigh, skinfold thickness of subscapular, time of exposure to cART _months_, body circumference of chest, and race _(Asian)_ (“Yes” for Asian race = 1; “No” = 0).

**Conclusions:**

The proposed anthropometric models advance the field of public health by facilitating early diagnosis and better management of lipodystrophy, a serious adverse health effect experienced by PLWHA.

## Background

The human immunodeficiency virus (HIV) remains one of the most serious global health threats of our time. Global estimates from 2016 found that 36.7 million people are living with HIV. Low- and middle-income countries, primarily in Africa and Latin America, represent 27.5 million (75%) of the total people living with HIV worldwide [1].

A few decades ago, people living with HIV/AIDS (PLWHA) were given a short life expectancy. At the peak of the HIV epidemic between 1990 and 2000, life expectancy for PLWHA dropped to 49.5 years in some low- and middle-income countries; HIV and AIDS (acquired immunodeficiency syndrome) combined, decreased life expectancy by approximately 20 years [2]. The introduction of the combined antiretroviral therapy (cART) in 1996, however, greatly changed this trend. cART is the current standard treatment for HIV [3]. With cART, PLWHA become less susceptible to the development of tumors and opportunistic infections [4]. As a result of cART treatment, today the life expectancy of people living with HIV/AIDS has increased 37 years [5]. Local and international efforts have focused on access to cART for those in need. Currently, around 53% of all people living with HIV have access to treatment [1].

The improvement of the cART therapeutic regimen is one of the greatest public health achievements of the past few decades, and the HIV infection has transitioned into a manageable chronic illness [6]. In more recent years there has been a shift in research focus, from the HIV-related communicable diseases to its long-term impact on non-communicable diseases. This trend is supported by recent studies that have looked into HIV-related cardiovascular disease, kidney- and liver-related diseases, cognitive decline, and osteoporosis [7–11]. Additional adverse health effects are HIV-related metabolic and body composition alterations. This article focuses on HIV-associated body composition alterations. Such changes include lipohypertrophy, lipoatrophy, or both, and are known as the “lipodystrophy of HIV” [12]. Lipohypertrophy is characterized by an increase in fat mass in the abdomen (visceral), back of the neck, and breast (gynecomastia). Lipoatrophy is characterized by the loss of fat mass in subcutaneous areas of the face, buttocks, arms, and legs [13].

Lipodystrophy is associated with an increased risk for the development of non-communicable diseases among PLWHA. The literature has suggested that PLWHA under cART treatment with lipodystrophy—mainly those with visceral fat accumulation—are at increased risk of developing cardiovascular diseases [14–17] and diabetes [18]. The association of lipodystrophy with non-communicable disease, especially atherosclerosis, is due to lipodystrophy’s negative effects on lipids and insulin sensitivity, and pro-inflammatory effects on endothelial cells [19, 20]. Thus, an earlier diagnosis and treatment for lipodystrophy can prevent or delay the development of some non-communicable diseases, thereby leading to better overall health and well-being of PLWHA, while reducing costs of treatment and premature deaths [21]. Additional problems associated with lipodystrophy include social and psychological distress that may contribute to discontinuation of cART treatment [22].

Multiple factors are associated with the pathophysiology of lipodystrophy, including cART’s protease inhibitor (PI) that appears to accelerate the rate of development of body composition and metabolic alterations [23, 24]. Other related factors include HIV clinical stage, age at the start of cART, race, and exercise level [25, 26] Since women living with HIV/AIDS experience different metabolic alterations [27] and lipodystrophy characteristics [28] compared to men, sex differences are other important factors to consider when studying lipodystrophy.

The prevalence of lipodystrophy among people living with HIV/AIDS can range from 41 to 70% [29, 30]. This imprecise prevalence is partially due to the absence of a methodological consensus for diagnosing lipodystrophy. Cost and accuracy are major methodological challenges. A clinical evaluation, which is an inexpensive and subjective diagnosis, is commonly used to diagnose lipodystrophy [31]. However, this method fails to accurately assess body composition alterations, especially in early stages. The “gold standard” approach for diagnosis and monitoring of lipodystrophy is imaging methods such as magnetic resonance image, computed tomography, and dual energy X-ray absorptiometry (DXA). DXA is a less costly and more accessible alternative imaging method when compared to the former methods [30, 31]. Additional advantages of DXA include: non-invasive and safe since it involves a minimum amount of radiation. A recommended method to measure body composition [32], DXA uses high and low energy X-ray photons to assess the composition and quantification of fat in regional and whole-body measurements. Using information from regional fat mass from DXA, an index “fat mass ratio” (FMR) was proposed considering data of trunk and leg fat mass to diagnose lipodystrophy [33]. Specific cutoff points for FMR have been developed for specific populations living with HIV/AIDS [33–35]. The uses of FMR have advanced in the field of body composition assessment among PLWHA. However, although researchers agree that diagnosing lipodystrophy with FMR is the most accurate, DXA is not very accessible [36]. The high operational cost of imaging technologies is an important factor that limits their utilization in clinical settings and in studies with a large number of participants from low- and middle-income countries. This lack of consensus regarding accurate and low-cost methods for diagnosing lipodystrophy remains unresolved in the field. A simplified method for diagnosing lipodystrophy could have a significant impact in low- and middle-income countries where HIV prevalence and incidence are the highest. Also, a feasible method for diagnosing and monitoring lipodystrophy should be available not only in research and hospital settings, but also in local healthcare settings where most patients receive care and medication for HIV.

Predictive models using anthropometric methods such as skinfold thickness and body circumferences are typically used with the general population to measure body composition [37, 38] and could be valuable tools for the diagnosis and monitoring of lipodystrophy in PLWHA. Anthropometric methods have been validated in various populations, including in low- and middle-income countries, where they have proven to be easy-to-use at a low operational cost. However, there is no current literature that has explored the development of accurate and simplified anthropometric predictive models to diagnose lipodystrophy in PLWHA. Our study proposes to fill this gap in the literature by developing anthropometric models to predict and subsequently validate lipodystrophy diagnoses for men and women living with HIV/AIDS. Findings from this study may contribute to advancing global health and epidemiological research on the long-term impact of HIV treatment on non-communicable diseases.

## Methods

### Participants

We employed a cross-sectional study design to evaluate people diagnosed with HIV/AIDS that were under cART treatment at the University Hospital of Ribeirao Preto School of Medicine, University of Sao Paulo, Brazil (HC- FMRP- USP/ UETDI). The study was conducted from November 2013 to November 2014. During the period of the study, 1298 people living with HIV/AIDS were receiving treatment at the HC- FMRP- USP/ UETDI. We set up a study booth next to the waiting room of the hospital. Using convenience sampling, we invited all people living with HIV/AIDS that were waiting for an appointment with a health professional to join our study. A total of 125 people living with HIV/AIDS agreed to participate, but 19 patients did not meet the inclusion and/or exclusion criteria. The inclusion criteria for this study were: diagnosed with HIV/AIDS, aged between 18 and 69 years, being under cART treatment, and with or without physical signs of lipodystrophy. The exclusion criteria were: being treated for opportunistic diseases or cancer, use of ergogenic products which could cause body composition alterations, being pregnant, using prosthesis, amputated, or engaged in an exercise program sometime in the past 6 months. We were able to reach about 10% of the people living with HIV/AIDS in treatment at our hospital. Our final sample included 106 people (65 men and 41 women) living with HIV/AIDS.

### Procedures

A DXA scan allows for a non-invasive, accurate, and fast measurement of body composition. In our study, we used the DXA Hologic instrument model QDR 4500 W, which was operated by a trained technician following standard procedures [32]. We used the fat mass ratio (FMR) as the dependent variable for lipodystrophy diagnosis. FMR is the ratio between the percent of the trunk fat mass and the percent of the lower-limb fat mass measured by DXA [33]. To predict the FMR by DXA, we used the potential independent variables which included all anthropometric measurements (skinfold thickness, body circumferences), and anthropometric ratios (adjusted variables). Additional predictor variables were collected through a questionnaire including; age (years), race (Caucasian, Black, Brown, and Asian), formal education (years), time of HIV diagnosis (years since the diagnosis), time of cART (years since the beginning of treatment), and type of cART (if the patient uses protease inhibitor “PI” or not).

We collected anthropometric measurements of participants in order to establish anthropometric predictive models for the diagnosis of lipodystrophy. The measurements included body weight and height, which were respectively assessed by a Filizola® electronic anthropometric scale and a Filizola® aluminum stadiometer. Skinfold thickness (SK) was measured in six regions: triceps, subscapular, suprailiac, abdomen (horizontal), thigh, and medial calf. The Prime® compass (Harpenden Scientific model) was used to measure each participant’s skinfolds. The guidelines for carrying out the skinfold thickness measures are those described by Harrison et al. (1988) [39]. Body circumferences (BC) were measured at nineteen regions: shoulder (biggest diameter), breast (fourth costosternal joint), waist (lower diameter), abdomen (umbilical scar), hip (biggest diameter), right arm extended, right arm contracted, right forearm, right wrist, left arm extended, left arm contracted, left forearm, left wrist, right thigh (proximal), right medial calf (biggest diameter), right ankle (lower circumference from the ankle, nearest to malleolus), left thigh (proximal), left medial calf (bigger diameter), and left ankle (lower circumference from the ankle, nearest to malleolus). A 2 m Sanny® brand metal band with a latex device at the end was used, which was replaced after every 20 participants were evaluated. The guidelines for carrying out the body circumference measurements are those described by Callaway et al. (1988) [40]. The BC of right arm extended, right arm contracted, left thigh (proximal), and left medial calf (bigger diameter) were corrected for the corresponding segment of subcutaneous adipose tissue thickness [41]. We corrected the BC measurements using skinfold thickness measurements to control for the influence of muscles on our predictive models [42].

In addition to investigating if the combination of SK and/or BC can best predict the FMR, we created ratios, or “adjusted variables,” based on the literature on the measurement characteristics of lipohypertrophy and lipoatrophy [13]. The body circumference ratios suggested in our study for the diagnosis of lipodystrophy were as follows: BC waist to hip ratio (BC Waist/Hip), BC waist to thigh ratio (BC Waist/Thigh), BC waist to medial calf leg ratio, BC abdomen to hip ratio, and BC abdomen to arm extended ratio. The skinfold thickness (SK) ratios suggested were: SK subscapular to medial calf ratio (SK Sc/Mc), SK triceps to thigh ratio, SK suprailiac to triceps ratio, SK abdomen to thigh ratio, and sum SK trunk (abdomen + subscapular + suprailiac) to sum SK appendicular (triceps + thigh + medial calf) ratio (Sum SK Trunk/Apen).

To ensure the quality of our data we took three precautionary steps. First, all the anthropometric measurements listed above were collected by the same evaluator at three-time points, and the median values were used. In addition, the measurements were conducted with 6 patients at a time and they were re-tested an hour later to confirm accuracy. Finally, the technical error of measurement (TEM) was calculated. TEM values were assessed for body circumferences (TEM ≤0.71 cm) and skinfold thickness (TEM ≤0.60 mm), ensuring the reliability of the measurements within established limits [43].

### Statistical analysis

Prior to conducting data analysis, we checked the database and cleaned for errors of data entry and impossible/inconsistent values. Means and standard deviations were calculated for all variables by sex, with a confidence interval of 95%. The Kolmogorov-Smirnov test was used to examine the normality of the distribution of continuous variables. The Student t-test and U Mann Whitney test for independent variables were used to assess differences between men and women based on their anthropometric measurements and additional predictor variables.

To control for sex differences in body composition in people living with HIV/AIDS, we studied men and women separately. Our sample was classified into two groups, men and women, for data analysis and anthropometric predictive models for the diagnosis of lipodystrophy.

We chose to use principal component analysis to select the variables for the predictive model’s generation in an attempt to reduce the amount of variables while not jeopardizing the assumptions expected for predictions. A total of 50 variables, including additional predictor variables (*n* = 8), anthropometric ratios (*n* = 10), and all anthropometric variables (*n* = 32) were used in the principal components analysis. The Eigenvalues (> 1) and highest extraction value (> 0.90) were adopted as criteria for the inclusion of variables. The principal component analysis reduced the number of variables to eleven: BC of right arm extended, BC of right arm contracted, SK triceps, BC of abdomen to arm extended ratio, body weight, SK thigh, BC of right thigh, BC of left arm extended, sum SK trunk (abdomen + subscapular + suprailiac) to sum SK appendicular (triceps + thigh + medial calf) ratio, SK abdomen, and BC right arm contracted corrected. We then conducted the stepwise linear regression using the reduced number of variables for each sex. However, we did not find high values for the adjusted R^2^ for the predictive models in both sexes (data not shown).

Therefore, we conducted the stepwise linear regression including all the anthropometric variables, anthropometric ratios, and additional predictor variables due to their association with lipodystrophy. The stepwise linear regression-generated models were conducted separately for men and women to predict FMR measured by DXA.

The limits of VIF = < 4.0, and Eigenvalues = > 0.7 were adopted. The adjusted R^2^ value was shown for each predictive model. Bland-Altman plots were used to explore distributions of errors. We developed binary (dummy) variables for the additional predictor variables ‘race’ and ‘type of cART’ to investigate their effect on the models. The standard error of the estimate (SEE) was used to define the accuracy of predictive models. To investigate limits of agreements between FMR predicted by DXA and FMR estimated by predictive models, 95% CI was used. The FMR estimated by predictive models is the outcome variable in this study. All the statistical analyses were carried out using SPSS® version 23.0 with a significance level of *p* < 0.05.

We conducted cross-validations by “leave and out” from the predicted residual error sum of squares (PRESS) method to measure the efficiency of each predictive model generated. The R^2^_PRESS_ and SEE _PRESS_ were expressed for all predictive models by sex. The PRESS analysis was carried out using Minitab® software version 17.

## Results

Average age of the participants was 46.2 ± 9.6 years, time of diagnosis of HIV 115.3 ± 80 months (9.6 years), and exposure to cART 87.4 ± 63.1 months (7.3 years). Formal education was 8.3 ± 3.5 years, 28% were smokers (*n* = 30), 24% consumed alcoholic drinks (*n* = 25), and 3% used illicit drugs (*n* = 4).

Results of the descriptive analysis by sex (Table 1) showed homogeneity of participants among age (*p* = 0.966), diagnosis of HIV (*p* = 0.387), and exposure to cART treatment (*p* = 0.306). We observed similar proportions (%) of self-reported race/ethnicity between men and women: White (69.2 / 53.6), Black (7.7 / 12.2), Asian (6.2 / 12.2), and Brown (16.9 / 22.0), respectively. Men used cART with-protease inhibitor (PI) (47.7%) and without-PI (52.3%), and women used cART with-PI (36.6%) and without-PI (63.4%). Men had on average three more years of formal education than women (*p* = 0.011).

**Table 1:** Descriptive analysis and differences test in people living with HIV/AIDS in Ribeirao Preto, Brazil

Variables	Participants (*n* = 106)				Differences test	*p* value
	Men (n = 65)		Women (*n* = 41)			
	Mean ± SD	95% CI	Mean ± SD	95% CI		
Age (years)	46.3 ± 8.9	44.2 to 48.5	46.2 ± 11.2	42.9 to 49.9	0.0	0.966
DXA-Total body weight (kg)	70.7 ± 11.5	67.8 to 73.4	63.6 ± 11.6	59.7 to 67.1	3.0	0.003*
DXA-Total trunk fat (kg)	26.0 ± 6.3	24.4 to 27.5	33.8 ± 7.0	31.4 to 35.9	−5.8	< 0.001
DXA-Total legs fat (kg)	5.2 ± 2.3	4.6 to 5.8	8.1 ± 3.6	7.0 to 9.2	−4.9	< 0.001*
DXA-FMR	1.2 ± 0.3	1.1 to 1.3	0.9 ± 0.2	0.9 to 1.0	5.2	< 0.001*
Height (cm)	170.3 ± 6.5	168.7 to 172.1	156.0 ± 5.8	154.2 to 158.0	11.5	< 0.001*
Diagnosis of HIV (months)	119.6 ± 74.8	100.5 to 137.6	105.5 ± 86.0	79.3 to 131.9	0.9^a^	0.387
Exposure to cART (months)	92.6 ± 63.2	76.0 to 107.9	79.4 ± 63.0	60.2 to 99.7	1.0^a^	0.306
Formal Education (years)	9.0 ± 3.6	8.0 to 9.8	7.3 ± 2.9	6.4 to 8.2	2.6^a^	0.011*
White (n/%)	45 (69.2)		22 (53.6)		–	–
Black (n/%)	5 (7.7)		5 (12.2)		–	–
Asian (n/%)	4 (6.2)		5 (12.2)		–	–
Brown (n/%)	11 (16.9)		9 (22.0)		–	–
Type of cART (With-PI) (n/%)	31 (47.7)		15 (36.6)		–	–
Type of cART (Without-PI) (n/%)	34 (52.3)		26 (63.4)		–	–
**Anthropometric Measurements**						
***Body Circumferences (cm)***						
Left arm extended	28.7 ± 3.0	28.0 to 29.5	28.0 ± 3.8	26.8 to 29.1	1.2	0.255
Left arm contracted	29.6 ± 2.9	28.9 to 30.4	29.0 ± 3.8	27.8 to 30.1	0.9	0.359
Left forearm	26.2 ± 1.8	25.8 to 26.6	23.9 ± 2.2	23.2 to 24.5	5.8	< 0.001*
Left wrist	16.7 ± 2.0	16.2 to 17.2	15.7 ± 1.4	15.2 to 16.1	3.0	0.004*
Right arm extended	29.1 ± 3.0	28.4 to 29.8	28.7 ± 3.8	27.5 to 29.8	0.6	0.526
Right arm extended corrected	26.5 ± 2.3	26.0 to 27.1	23.3 ± 2.2	22.6 to 24.0	7.1	< 0.001*
Right arm contracted	30.2 ± 2.8	29.6 to 30.9	29.6 ± 3.8	28.4 to 30.7	1.0	0.335
Right arm contracted corrected	27.6 ± 2.3	27.1 to 28.2	24.2 ± 2.3	23.5 to 24.9	7.5	< 0.001*
Right forearm	26.7 ± 1.8	26.3 to 27.2	24.4 ± 2.1	23.7 to 25.0	6.0	< 0.001*
Right wrist	16.7 ± 1.0	16.4 to 16.9	15.7 ± 1.4	15.3 to 16.1	3.9	< 0.001*
Left thigh	49.8 ± 8.5	47.4 to 51.7	54.2 ± 10.0	50.7 to 57.1	−2.4	0.016*
Left medial calf	35.0 ± 2.7	34.3 to 35.7	34.2 ± 3.9	32.9 to 35.4	1.3	0.214
Left ankle	21.7 ± 1.5	21.3 to 22.1	20.9 ± 1.7	20.3 to 21.4	2.5	0.016*
Right thigh	51.7 ± 5.5	50.3 to 53.1	55.6 ± 7.9	52.9 to 58.0	−2.9	0.004*
Right thigh corrected	47.9 ± 4.4	46.8 to 49.1	47.9 ± 5.4	46.2 to 49.6	0.0	0.977
Right medial calf	35.0 ± 2.8	34.3 to 35.7	34.3 ± 4.0	33.0 to 35.4	1.1	0.283
Right medial calf corrected	33.2 ± 2.6	32.5 to 33.9	29.9 ± 2.9	29.0 to 30.8	6.0	< 0.001*
Right ankle	21.7 ± 1.4	21.3 to 22.0	20.7 ± 1.6	20.2 to 21.2	3.1	0.003*
Shoulder	108.4 ± 6.8	106.6 to 110.0	100.4 ± 7.6	97.8 to 102.8	5.5	< 0.001*
Breast	97.9 ± 6.8	96.1 to 99.4	85.9 ± 7.0	83.8 to 88.2	8.5	< 0.001*
Waist	89.05 ± 8.9	86.7 to 91.2	83.7 ± 8.7	80.8 to 86.3	3.0	0.003*
Abdomen	90.4 ± 13.5	86.8 to 93.3	92.4 ± 10.3	89.3 to 95.6	−0.8	0.409
Hip	91.8 ± 7.5	90.0 to 93.6	97.9 ± 10.3	94.5 to 100.9	−3.5	0.001*
Waist/Hip ratio	1.0 ± 0.1	1.0 to 1.1	0.9 ± 0.1	0.8 to 0.9	6.9	< 0.001*
Waist/Thigh ratio	1.8 ± 0.3	1.7 to 1.9	1.6 ± 0.2	1.5 to 1.6	4.4	< 0.001*
Waist/Medial calf leg ratio	2.6 ± 0.2	2.5 to 2.6	2.5 ± 0.3	2.4 to 2.6	1.8	0.071
Abdomen/Hip ratio	1.0 ± 0.1	1.0 to 1.0	1.0 ± 0.1	1.0 to 1.0	2.1	0.043*
Abdomen/Arm extended ratio	3.3 ± 0.5	3.2 to 3.4	3.6 ± 0.3	3.5 to 3.7	−4.5	< 0.001*
***Skinfold Thickness (mm)***						
Triceps	8.3 ± 4.5	7.3 to 9.5	17.3 ± 8.0	14.6 to 19.7	−7.2	< 0.001*
Subscapular	18.8 ± 7.5	16.7 to 20.7	23.4 ± 8.9	20.5 to 26.2	−2.8	0.006*
Suprailiac	15.6 ± 8.7	13.4 to 17.7	24.5 ± 9.1	21.5 to 27.2	−5.0	< 0.001*
Horizontal abdomen	19.0 ± 8.7	17.0 to 21.2	24.8 ± 8.8	22.0 to 27.5	−3.3	0.002*
Thigh	11.9 ± 8.4	9.9 to 14.1	24.4 ± 11.1	20.7 to 27.6	−6.4	< 0.001*
Medial calf	5.7 ± 4.5	4.6 to 7.0	14.0 ± 7.6	11.5 to 16.3	−6.9	< 0.001*
Subscapular/Medial calf ratio	4.8 ± 3.6	4.0 to 5.7	3.2 ± 4.6	2.0 to 4.8	2.0	0.044*
Suprailiac/Triceps ratio	2.0 ± 1.0	1.8 to 2.3	1.6 ± 0.6	1.4 to 1.8	3.0	0.004*
Abdomen/Thigh ratio	2.0 ± 1.0	1.8 to 2.3	1.3 ± 1.0	1.0 to 1.6	3.7	< 0.001*
Sum Sk Trunk/Apen	2.4 ± 1.0	2.2 to 2.7	1.6 ± 0.8	1.3 to 1.8	4.7	< 0.001*

Note: *HIV* Human Immunodeficiency virus, *Aids* Acquired immunodeficiency syndrome; 95% CI: Confidence interval; Signal ^a^Mann Whitnney test; Signal *: p < 0,05; *FMR* Fat Mass Ratio, *DXA* Dual Energy X-ray Absorptiometry, *cART* Combined Antiretroviral Therapy, *PI* Protease Inhibitor, *Sum SK Trunk/Apen* Sum of Skinfold thickness of Trunk (abdomen+subscapular+suprailiac) to sum appendicular Skinfold Thickness (triceps+thigh+medial calf) ratio

For the anthropometric measurements, there were no statistical differences between sexes in most body circumferences (BC): left arm extended and contracted (*p* = 0.255 and *p* = 0.359, respectively) right arm extended and contracted (*p* = 0.526 and *p* = 0.335), left medial calf (*p* = 0.214), right thigh corrected (*p* = 0.977), right medial calf (*p* = 0.238), abdomen (*p* = 0.409), and ratio of waist/medial calf (*p* = 0.071). Regarding DXA, we observed differences in body composition among sex for all variables, where men have a higher body weight (kg) and FMR values, (*p* = 0.003, *p* < 0.001, respectively), and lower total trunk fat (kg) (p < 0.001) and total leg fat (kg) (p < 0.001).

### Predictive models to estimate fat mass ratio

After stepwise linear regression adjusted and non-adjusted, predictive models were proposed for men and women. Details of each predictive model, validation, and errors estimates from FMR by DXA and FMR by predictive models are presented below.

### Predictive models for men:

Table 2 shows 11 (6 adjusted and 5 non-adjusted) predictive models to estimate FMR for men. After conducting stepwise linear regressions for the adjusted predictive models, we observed variations of the adjusted R^2^ value between 0.50 and 0.77, of the SEE between 0.14 and 0.20, and of the 95% CI between 1.15 and 1.32. The adjusted R^2^ value was observed between 0.34 and 0.70, the SEE between 0.16 and 0.23, and the 95% CI between 1.15 and 1.29 for non-adjusted predictive model variations.

**Table 2:** Adjusted and non-adjusted predictive models for lipodystrophy diagnosis and validation for 65 men living with HIV/AIDS

Models		Independents variables									Validation	
Men adjusted	Sk Sc/Mc	Sk Thigh	BC Waist	Formal Education _years_	T HIV _months_	Type cART _WI/PI = 1 WO/PI = 0_	β	r^2^ adjust	SEE	95%CI	r^2^_PRESS_	SEE _PRESS_
1	0.056 ± 0.007						0.93 ± 0.04	0.50	0.20	1.16 to1.26	0.43	0.22
2	0.043 ± 0.007	−0.011 ± 0.003					1.12 ± 0.07	0.58	0.19	1.15 to 1.26	0.52	0.20
3	0.034 ± 0.007	−0.016 ± 0.003	0.011 ± 0.003				0.27 ± 0.21	0.67	0.16	1.17 to 1.28	0.65	0.17
4	0.031 ± 0.006	−0,018 ± 0.003	0.011 ± 0.002	0.017 ± 0.006			0.14 ± 0.20	0.71	0.15	1.18 to 1.30	0.70	0.16
5	0.027 ± 0.006	−0.019 ± 0.003	0.011 ± 0.002	0.020 ± 0.005	0.001 ± 0.000		0.07 ± 0.19	0.74	0.15	1.19 to 1.32	0.71	0.16
6	0.029 ± 0.006	−0.017 ± 0.003	0.010 ± 0.002	0.020 ± 0.005	0.001 ± 0.000	−0.094 ± 0.036	0.13 ± 0.19	0.77	0.14	1.15 to 1.27	0.73	0.15
Men non-adjusted	Sk Thigh	BC Waist	Formal Education _years_	T HIV _months_	BC Left Leg							
1	−0.020 ± 0.004						1.44 ± 0.05	0.34	0.23	1.16 to 1.25	0.30	0.24
2	−0.024 ± 0.003	0.015 ± 0.003					0.17 ± 0.25	0.54	0.19	1.17 to 1.27	0.52	0.20
3	−0.026 ± 0.003	0.015 ± 0.003	0.021 ± 0.006				0.02 ± 0.24	0.61	0.18	1.18 to 1.29	0.60	0.18
4	−0.025 ± 0.003	0.014 ± 0.002	0.024 ± 0.006	0.001 ± 0.000			−0.05 ± 0.22	0.66	0.17	1.17 to 1.29	0.64	0.17
5	−0.023 ± 0.003	0.017 ± 0.003	0.027 ± 0.006	0.001 ± 0.000	−0.023 ± 0.010		0.41 ± 0.29	0.70	0.16	1.15 to 1.26	0.66	0.17

Note: *HIV* Human Immunodeficiency virus, *Aids* Acquired immunodeficiency syndrome, *Sk Sc/Mc* Skinfold thickness of subscapular to medial calf ratio, *Sk Thigh* Skinfold Thickness of Thigh, *BC Waist* Body circumference of Waist, *T HIV*_*months*_ time of diagnosis to HIV in months; Type cART WI/PI = 1 WO/PI = 0 = type of combined therapy antiretroviral. With Protease Inhibitor presence = 1 and without Protease Inhibitor presence = 0; BC Left Leg: Body circumference of Left Leg; β = Beta value; r^2^ adjust = r square adjusted; SEE = standard error of estimate; CI 95% = confidence interval; r^2^_PRESS:_ r square _PRESS;_ SEE _PRESS:_ standard error of estimate _PRESS_

#### Best predictive model for men

We considered adjusted model 6 to be “the best” predictive model because it includes few independent variables and a higher power of prediction. Linear regression resulted in a high adjusted R^2^ value of 0.77, a SEE value of 0.14, and a 95% CI value of 1.15 to 1.27. The variables included were: ratio of skinfold thickness of subscapular to medial calf, skinfold thickness of thigh, body circumference of waist, formal education _years_, time of diagnosis to HIV _months_, and type of cART (with PI “WI/PI = 1” or without PI “WO/P1 = 0”).

$$ {\displaystyle \begin{array}{l}\mathbf{Fat}\ \mathbf{Mass}\ \mathbf{Ratio}=0.134+\left(\left(\mathrm{R}\ \mathrm{Sk}\ {\mathrm{Sc}}_{\left[\mathrm{mm}\right]}/\mathrm{Sk}\ {\mathrm{Mc}}_{\left[\mathrm{mm}\right]}\right)\times 0.029\right)+\left(\mathrm{Sk}\ {\mathrm{Thigh}}_{\left[\mathrm{mm}\right]}\times -0.017\right)\\ {}+\left(\mathrm{BC}\ {\mathrm{Waist}}_{\left[\mathrm{cm}\right]}\times 0.010\right)+\left({\mathrm{Formal}\ \mathrm{Education}}_{\left[\mathrm{years}\right]}\times 0.020\right)+\left(\mathrm{T}\ {\mathrm{HIV}}_{\left[\mathrm{months}\right]}\times 0.001\right)\ \\ {}+\left({\mathrm{Type}\ \mathrm{cART}}_{\left[\mathrm{WI}/\mathrm{PI}=1\ \mathrm{WO}/\mathrm{PI}=0\right]}\times -0.094\right)\end{array}} $$ *(Men adjusted model 6)*

**Fig. 1:**
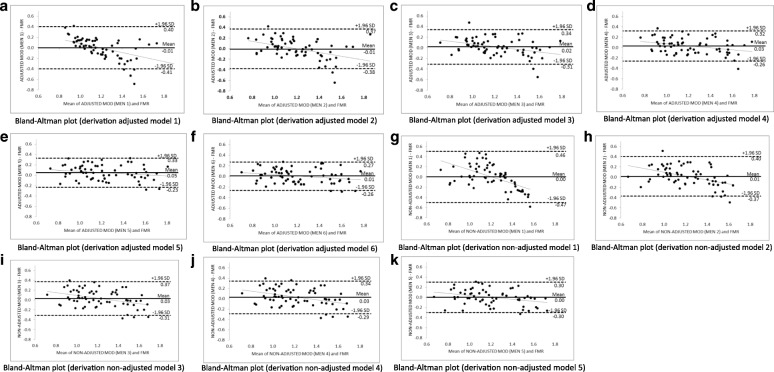
Bland-Altman plots for derivation adjusted and non-adjusted (**a**-**k**) predictive models (MOD) of fat mass ratio (FMR) for men living with HIV/AIDS

Figure 1 shows the Bland-Altman plots for each adjusted (a, b, c, d, e, and f) and non-adjusted (g, h, i, j, and k) predictive model for men. The agreement between the FMR by DXA and FMR by adjusted predictive models were accurate, since the models exhibited practically no bias (− 0.01 until + 0.05), with limits of agreement reduced, especially for the best model for men (− 0.26 and + 0.27). In addition, it was found that there was a small tendency for predictive models to underestimate the FMR by DXA when the FMR values were higher, and to overestimate when the FMR values were lower. However, there was an accurate use of the predictive models to diagnose lipodystrophy, since the extreme values of FMR were rarely outside the limits of agreement.

### Predictive models for women:

Table 3 shows 10 (5 adjusted and 5 non-adjusted) predictive models to estimate FMR for women. After stepwise linear regression for adjusted predictive models, variations of the adjusted R^2^ values were observed between 0.69 and 0.83, of the SEE between 0.10 and 0.13, and of the 95% CI between 0.87 and 1.06. For non-adjusted predictive models we observed variations of the adjusted R^2^ values between 0.27 and 0.78, of the SEE between 0.11 and 0.20, and of the 95% CI between 0.81 and 1.03.

**Table 3:** Adjusted and non-adjusted predictive models for lipodystrophy diagnosis and validation for 41 women living with HIV/AIDS

Models	Independents variables									Validation	
Women adjusted	Sum Sk Trunk/Appen	BC R Arm ext. c	BC Waist/Hip	T HIV _months_	BC Waist/Thigh	β	r^2^ adjust	SEE	95%CI	r^2^_PRESS_	SEE _PRESS_
1	0.229 ± 0.025					0.58 ± 0.04	0.69	0.13	0.88 to 1.00	0.63	0.14
2	0.222 ± 0.022	0.028 ± 0.008				−0.07 ± 0.20	0.75	0.11	0.87 to 1.00	0.71	0.12
3	0.192 ± 0.024	0.027 ± 0.008	0.634 ± 0.255			−0.54 ± 0.26	0.79	0.11	0.88 to 1.00	0.75	0.11
4	0.169 ± 0.024	0.026 ± 0.007	0.683 ± 0.239	0.001 ± 0.000		−0.57 ± 0.25	0.81	0.10	0.92 to 1.06	0.77	0.11
5	0.199 ± 0.027	0.019 ± 0.008	1.109 ± 0.296	0.001 ± 0.000	−0.255 ± 0.115	−0.43 ± 0.24	0.83	0.10	0.84 to 0.96	0.80	0.10
Women non-adjusted	Sk Thigh	Sk Subscapular	T cART _months_	BC Breast	Race _(Asian)_						
1	−0.011 ± 0.003					1.21 ± 0.08	0.27	0.20	0.91 to 0.99	0.17	0.20
2	−0.015 ± 0.002	0.017 ± 0.003				0.91 ± 0.07	0.66	0.13	0.90 to 1.02	0.62	0.14
3	−0.013 ± 0.002	0.016 ± 0.002	0.001 ± 0.000			0.82 ± 0.07	0.71	0.13	0.91 to 1.03	0.64	0.13
4	−0.013 ± 0.002	0.010 ± 0.003	0.001 ± 0.000	0.010 ± 0.004		0.08 ± 0.29	0.75	0.12	0.89 to 1.01	0.68	0.13
5	−0.011 ± 0.002	0.008 ± 0.003	0.001 ± 0.000	0.012 ± 0.004	−0.147 ± 0.062	−0.15 ± 0.29	0.78	0.11	0.81 to 0.93	0.71	0.12

Note: *HIV* Human Immunodeficiency virus, *Aids* Acquired immunodeficiency syndrome, *Sum SK Trunk/Appen* Sum of Skinfold thickness of Trunk (abdomen+subscapular+suprailiac) to sum appendicular Skinfold Thickness (triceps+thigh+medial calf) ratio, *BC R Arm ext c* Body circumference of right arm extended corrected, *BC Waist/Hip* Body circumference waist to hip ratio, *T HIV*_*months*_ time of diagnosis to HIV in months, *BC Waist/Thigh* Body circumference of waist to thigh ratio, *SK Thigh* Skinfold Thickness of thigh, *SK Subscapular* Skinfold Thickness of subscapular, *T cART*_*months*_ Time of exposure to combined antiretroviral therapy in months, *BC Breast* Body circumference of breast; Race _(Asian)_: yes = 1 for race Asian and no = 0 for race Asian; β = Beta value; r2 adjust = r square adjusted; SEE = standard error of estimate; CI 95% = confidence interval; r^2^ PRESS: r square PRESS; SEE PRESS: standard error of estimate PRESS

#### Best predictive model for women

We considered non-adjusted model 5 to be “the best” predictive model for women because it includes few independent variables and a high power of prediction. From the linear regression, we had a high adjusted R^2^ value of 0.78, a SEE value of 0.11, and a 95% CI value of 0.81 to 0.93. The variables included were: skinfold thickness of thigh, skinfold thickness of subscapular, time of exposure to cART _months_, body circumference of chest, and race _(Asian)_ (“Yes” for Asian race = 1; “No” = 0).

$$ {\displaystyle \begin{array}{l}\mathbf{Fat}\ \mathbf{Mass}\ \mathbf{Ratio}=-0.15+\left(\mathrm{Sk}\ {\mathrm{Thigh}}_{\left[\mathrm{mm}\right]}\times -0.011\right)+\left(\mathrm{Sk}\ {\mathrm{Subscapular}}_{\left[\mathrm{mm}\right]}\times 0.008\right)\ \\ {}+\left(\mathrm{T}\ {\mathrm{cART}}_{\left[\mathrm{months}\right]}\times 0.001\right)+\left(\mathrm{BC}\ {\mathrm{Breast}}_{\left[\mathrm{cm}\right]}\times 0.012\right)\ \\ {}+\left({\mathrm{Race}}_{\left(\mathrm{Asian}\right)\left[\mathrm{yes}=1;\mathrm{no}=0\right]}\times -0.147\right)\end{array}} $$ *(Women non-adjusted model 5)*

**Fig. 2:**
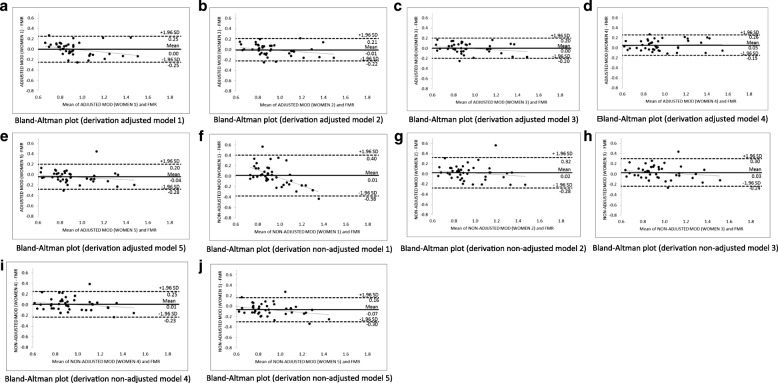
Bland-Altman plots for derivation adjusted and non-adjusted (**a**-**j**) predictive models (MOD) of fat mass ratio (FMR) for women living with HIV/AIDS

Figure 2 shows the Bland-Altman plots for each adjusted (a, b, c, d, and e) and non-adjusted (f, g, h, i, and j) predictive models for women. Our analysis shows accurate agreement between the FMR by DXA and FMR by predictive models, since the models exhibited practically no bias (− 0.07 until + 0.05), with limits of agreement reduced, especially for the best model for women (− 0.30 and + 0.16). In addition, there was a small tendency of predictive models to underestimate the FMR by DXA when the FMR values were higher and to overestimate when the FMR values were lower. Only adjusted predictive model 4 shows the opposite trend. However, all models were accurate for the smallest and highest values of FMR, since no estimate for these values was outside the limits of agreement.

### Validation of predictive models to predict fat mass ratio

We validated the adjusted and non-adjusted predictive models to estimate FMR. For our best predictive model for men (adjusted model 6) (Table 2), we had a high adjusted R^2^_PRESS_ value of 0.73, and a small SEE _PRESS_ value of 0.15. For the best predictive model for women (non-adjusted model 5) (Table 3), we also had a high adjusted R^2^value _PRESS_ of 0.71, and a small SEE _PRESS_ value of 0.12. The short interval for the limit of agreement observed in each predictive model by Bland-Altman plots and the adjusted R^2^_PRESS_ value together support the accuracy of these sex-specific predictive models for the diagnosis of lipodystrophy in people living with HIV/AIDS.

## Discussion

The main goal of this study was to predict and validate methods to diagnose lipodystrophy among people living with HIV/AIDS through anthropometric measurements and using DXA as a reference method. Prediction models were validated for both men and women. Our predictive models advance the field of HIV/AIDS studies by proposing a method for the diagnosis of lipodystrophy that is objective, accurate, low cost, and easy to use. In public health, especially in low- and middle-income countries, our predictive models should lead to improvements in the understanding of the prevalence, incidence, risk factors, pathogenesis, prevention, and treatment of lipodystrophy. In addition, the feasible identification of body composition alterations of lipodystrophy will contribute to interventions from health professionals that lead to better health outcomes on the overall well-being of people living with HIV/AIDS. To date, only France [33], Portugal [34], and Brazil [35, 44] have developed cutoff points for lipodystrophy diagnosis using fat mass ratio by DXA. Therefore, researchers and other health professionals using our predictive models could adopt the most convenient cutoff point for fat mass ratio by DXA as previously cited, or establish a cutoff point for our models to diagnose and monitor lipodystrophy among people living with HIV/AIDS.

Our study is important not only in the context of Brazil—place of residence of study participants—but to other countries with limited resources available to develop such studies, including Eastern and Southern African countries, the regions hit hardest by HIV/AIDS. To ensure global dissemination and accessibility, lipodystrophy diagnosis based on our predictive models can be found in an excel file in the following link (http://www2.eerp.usp.br/pos/arquivos/Routine_Models_Men_and_Women.xls).

Lipodystrophy is the second most common adverse health effect experienced by people living with HIV/AIDS undergoing cART treatment [45]. For this reason, special attention has been given to changes in body composition when treating HIV/AIDS patients. The development of the predictive models for lipodystrophy proposed by this study include important key variables such as age, time of diagnosis to HIV and exposure to cART, and type of cART treatment. These variables were controlled for both sexes to ensure true interpretations on each predictive model. Overall, the accuracy of the models was high as indicated by the adjusted R^2^ values found in both adjusted and non-adjusted predictive models in men and women.

Our results support the assumption that the best predictive model for men is ‘adjusted model 6,’ where the following anthropometric variables were included: ratio of skinfold thickness of subscapular to medial calf, skinfold thickness of thigh, and waist circumference. A recent study published by Alencastro et al. (2017) [36] compared self-reported signs of lipodystrophy (lipohypertrophy and lipoatrophy) with skinfold thickness and body circumferences among 815 people living with HIV/AIDS. The authors observed several associations in self-reported signs of lipodystrophy with objective measurements mainly of the increase of fat in the back of the neck and waist, and the decrease of fat in limbs. Those areas match with the variables included in our ‘adjusted model 6’ (for men), supporting their use to diagnose lipodystrophy. Moreover, the aforementioned risk factors are associated with the development of atherosclerotic cardiovascular disease, [25, 26] reinforcing the importance of an early and accurate diagnosis of lipodystrophy to prevent non-communicable diseases.

In addition, ‘adjusted model 6’ included important variables such as “education,” “time of diagnosis to HIV,” and “type of cART,” which are important social and physiological determinants for body composition alterations. Our findings on the associations between these factors and the development of lipodystrophy in people living with HIV/AIDS are supported by two systematic reviews [46, 47]. The reviews report evidence that low education level is associated with several adverse health outcomes among people living with HIV/AIDS, including low health knowledge and an increase in the incidence of chronic illness. The inclusion of the “education” variable in the model proposed by the current study underscores the association of high education level and healthy lifestyle with the prevention of lipodystrophy. A cohort study explored the effect of HIV infection in metabolic parameters in 419 antiretroviral-naïve HIV infected patients. The results showed—in the absence of cART treatment—that HIV disease influences lipid values and glucose homeostasis [48]. Time of diagnosis to HIV is the most influential variable because it expresses the effect of the virus’ influence on body composition and metabolic alterations over time. Two large studies provided evidence of the influence and type of cART treatment that causes the development of lipodystrophy. Martinez et al. (2001) [49] enrolled 494 people living with HIV/AIDS and, using clinical observation, investigated the risk factors for lipodystrophy after a three-year follow-up period and found that cART treatment including protease inhibitors is associated with the development of body composition alterations in lipodystrophy. In a cross-sectional study, Miller et al. (2003) [50] used DXA to evaluate 1348 people living with HIV/AIDS in different regions of Australia to establish the prevalence and risk factors associated with lipodystrophy. They found people living with HIV/AIDS and under cART treatment with protease inhibitors have the highest prevalence and are at the greatest risk of developing lipodystrophy. The literature above is in agreement with our findings. Our study developed predictive models for lipodystrophy diagnosis considering the influence of cART treatment by creating a dummy variable for protease inhibitors.

For women, we believe that the best predictive model is ‘non-adjusted model 5’. For this model, only three anthropometric variables were included: skinfold thickness of thigh, skinfold thickness of subscapular, and body circumference of breast. Zulfa et al. (2016) [51] used anthropometric measurements and DXA to describe changes in body fat distribution in a 24-month longitudinal study enrolling 132 women with HIV/AIDS. They observed that women showed a significant decrease in leg fat (%) from 45.2 to 42.1 and an increase in trunk fat (kg) from 7.6 to 11.7 after the follow-up. In addition, in a longitudinal study enrolling 922 people living with HIV/AIDS, Scherzer et al. (2011) [52] determined the association for all causes of mortality by measuring total and regional adipose tissue levels using imaging methods with a 5-year follow-up. After adjusting for multiple variables they found that an increase in trunk fat corresponds to an absolute risk of one death per 100 people living with HIV/AIDS over 5 years. These findings support the importance of the anthropometric variables included in our ‘non-adjusted model 5’ for women, due to the representativeness of lipodystrophy characteristics and association with non-communicable diseases and risk of death. Moreover, this model included variables such as “time of exposure to cART” and “race (Asian),” which reflect the influence of the time of treatment and genetic background on the development of lipodystrophy. The studies cited above suggest that protease inhibitors are important in the development of body composition alterations in people living with HIV/AIDS, and that the length of exposure to cART treatment is an influential factor for considering the severity of lipodystrophy [49, 50]. Regarding the association of race and lipodystrophy, there is no consensus on whether race has an influence on body composition alterations. In their review, Gripson and Carr (2005) [53] agree that lipodystrophy has multifaceted origins, but they do not show evidence of a distinct racial influence. Also, two studies found different associations regarding race. In a case-controlled study, Filippini et al. (2006) [54] assessed body composition to identify racial factors related to lipodystrophy’s prevalence in Sub-Saharan Black Africans and White Italians living with HIV/AIDS. They observed that lipodystrophy is more prevalent in White Italians (65.2%) than in Sub-Saharan Black Africans (4.4%). Andany et al. (2011) [55] investigated the influence of race on lipodystrophy characteristics in a cross-sectional study enrolling 778 people living with HIV/AIDS. After a multi-variable regression analysis was conducted, black women were found to be most vulnerable to lipodystrophy, mainly lipohypertrophy. We did not find in the literature a specific association with the Asian race for the development of lipodystrophy. In addition, in our model “race (Asian)^”^ is a dummy variable. Dichotomizing the variable as presence of “race (Asian)” or no presence provided us with accurate predictive power.

This study represents a first step towards proposing an objective, accurate, and low operational cost tool for lipodystrophy diagnosis and monitoring in men and women living with HIV/AIDS. We believe that the greatest challenge in the future is developing accessible predictive models separately for lipoatrophy and lipohypertrophy. This next step will enable better comprehension of the pathways that contribute to losses and gains in fat mass, and promotion of specific treatments (pharmacologic or otherwise) for each. Also, further analysis is needed to establish whether the predictive models for lipodystrophy differ between people living with HIV/AIDS in treatment for opportunistic disease, for those who are over 69 years old, and for those who are younger. The use of our findings in future studies of HIV/AIDS will allow for more reliable estimates of lipodystrophy prevalence, incidence, and cause, and more effective prevention and treatment strategies.

Findings should be interpreted with caution. Due to the amount of independent variables to estimate fat mass ratio and the separation of the sample into men and women, there are limitations regarding sample size of this study. We have chosen to separate men and women due to the similar number of people living with HIV/AIDS for both sexes in our total sample size. In addition, the body composition differences among sexes is established in the literature and confirmed in our results. In accordance with statistical assumptions, we reduced the number of independent variables through the principal component analysis. However, after we conducted stepwise linear regression adopting the reduced number of independent variables, we did not have predictive models with accurate predictive power for lipodystrophy diagnosis in men and women. In this case we assumed the inclusion of all additional predictor variables, anthropometric ratios, and all anthropometric variables.

The power of prediction based on the adjusted R^2^ values for our best predictive model for men (77) and women (78) is limited, but is representative of the complex factors which influence the high variability in body composition alterations in people living with HIV/AIDS. While the accuracy and significance could be greater, a prediction of 80% for lipodystrophy diagnosis can be achieved using an accessible and low operational cost tool that represents a great advancement in the health care services available to people living with HIV/AIDS.

In our study we did not exclude people living with HIV/AIDS with diabetes mellitus or other chronic metabolic diseases. These conditions are often experienced by people living with HIV/AIDS under cART treatment. Even though some chronic metabolic diseases may influence body composition alterations, our goal was to study the most typical group of people living with HIV/AIDS. However, we understand that the presence of diabetes mellitus or other chronic metabolic diseases could have influenced our predictive models.

## Conclusion

Accurate and sex-specific predictive models using anthropometric measurements were proposed to diagnose lipodystrophy in people living with HIV/AIDS. We believe this information will advance the public health field by assisting health professionals in diagnosing and monitoring lipodystrophy at a low cost, and thus will contribute to decreasing the adverse health effects of lipodystrophy in people living with HIV/AIDS.

## Abbreviations

BC: Body circumferences
cART: Combined antiretroviral therapy
DXA: Dual energy X-ray absorptiometry
FMR: Fat mass ratio
HC- FMRP- USP/UETDI: University Hospital of Ribeirao Preto School of Medicine, University of Sao Paulo, Brazil
HIV: Human immunodeficiency virus
PI: Protease inhibitor
PLWHA: People living with HIV/AIDS
PRESS: Predicted residual error sum of squares
SEE: Standard error of the estimate
SK: Skinfold thickness
TEM: Error of measurement
